# Distinct patterns of vascular failure in COL1A1-associated osteogenesis imperfecta and FBN1-associated Marfan syndrome

**DOI:** 10.3389/fcvm.2026.1922704

**Published:** 2026-10-27

**Authors:** Hiroshi Kubota, Hidehito Endo, Masao Nunokawa, Toshihiko Isaji, Sachito Minegishi, Hiroshi Tsuchiya, Masaru Kimura, Toru Ikezoe, Yusuke Inaba, Yu Takahashi, Kouta Kogure, Tomohiro Iwakura, Yaeko Ichikawa, Yutaka Hosoi

**Affiliations:** 1 Department of Cardiovascular Surgery, Kyorin University, Tokyo, Japan; 2 Department of Cardiovascular Surgery, Sakakibara Heart Institute, Tokyo, Japan; 3 Department of Neurology, Kyorin University, Tokyo, Japan

**Keywords:** aortic dissection, COL1A1, FBN1, genotype-phenotype correlation, heritable aortic disease, Marfan and associated disorders, osteogenesis imperfecta, vascular fragility

## Abstract

**Introduction:**

Heritable connective tissue disorders are an important cause of aortic disease; however, the mechanisms of vascular failure in genetically defined aortopathies remain incompletely understood.

**Methods:**

We analyzed the clinical course, imaging findings, and histopathologic features of two patients with clinically and genetically characterized connective tissue disorders: COL1A1-associated osteogenesis imperfecta and FBN1-associated Marfan syndrome.

**Results:**

The COL1A1-associated patient developed multilevel arterial complications, including aortic regurgitation requiring aortic valve replacement at age 36, rupture of a dissected right common iliac artery in the same year, chronic type A aortic dissection requiring root, ascending, and arch replacement at age 54, and rupture of the infrarenal abdominal aorta at a diameter of only 30 mm. The FBN1-associated patient presented with type B aortic dissection at age 34 and subsequently developed progressive aneurysmal disease requiring staged thoracoabdominal replacement, total arch replacement for acute type A dissection, and aortic root replacement, followed by multiple peripheral vascular reconstructions. Histopathologic examination demonstrated valvular myxomatous degeneration in both cases. The aortic wall in both patients showed medial degeneration with fragmentation of elastic fibers. In addition, the COL1A1-associated patient demonstrated abdominal aortic wall thinning, with a minimum thickness of 1.25 mm.

**Conclusions:**

These two patients exhibited contrasting longitudinal vascular courses despite some overlapping histopathologic findings. The COL1A1-associated OI patient experienced rupture of a dissected infrarenal aortic segment, whereas the FBN1-associated Marfan patient experienced repeated dissections followed by progressive vascular dilatation and multiple surgical reconstructions. These observations are descriptive and do not establish genotype-specific patterns or mechanisms of vascular failure.

## Introduction

Heritable connective tissue disorders represent an important subset of aortic disease. Marfan syndrome, caused by pathogenic variants in FBN1, is a prototypical aortopathy characterized by progressive aortic enlargement and risk of dissection.

In contrast, osteogenesis imperfecta (OI), most commonly caused by pathogenic variants in COL1A1 or COL1A2, is classically recognized as a disorder of bone fragility. However, type I collagen is a key structural component of the vascular wall, and cardiovascular involvement may be underrecognized. Although valvular abnormalities and aortic dilation have been described, clinically significant arterial rupture remains rare (1).

We report the longitudinal clinical, imaging, and available histopathologic findings of two genetically confirmed patients with COL1A1-associated osteogenesis imperfecta and FBN1-associated Marfan syndrome who exhibited contrasting vascular courses.

## Case presentation

### Case 1: Col1a1-associated osteogenesis imperfecta

A 57-year-old man had clinical features consistent with OI, including multiple fractures during childhood—specifically, bilateral femoral fractures at 6 months of age and a right lower leg fracture during early childhood (kindergarten age)—as well as blue sclerae, dentinogenesis imperfecta, hearing impairment, and generalized ligamentous laxity. Osteoporosis had been treated with bisphosphonate therapy. There was no family history of aortic disease.

At 36 years of age, the patient developed severe aortic regurgitation and underwent aortic valve replacement. Intraoperatively, the valve leaflets were markedly fragile and tore easily upon manipulation.

In the same year, rupture of a dissected right common iliac artery occurred, resulting in a large inguinal hematoma. Surgical fenestration and decompression were performed with good recovery.

At 54 years of age, follow-up imaging revealed chronic type A aortic dissection involving the aortic root, ascending aorta, and arch. The patient reported a prior episode of severe chest pain several months earlier, suggesting delayed diagnosis of acute dissection. Root, ascending, and total arch replacement were performed.

Later in the same year, rupture of the infrarenal abdominal aorta occurred distal to the origin of the inferior mesenteric artery. Intraoperative and imaging findings indicated that the rupture was associated with underlying aortic dissection rather than primary aneurysmal degeneration. Computed tomography performed five months earlier had demonstrated a maximal diameter of only 30 mm without significant aneurysmal dilatation.

Emergency abdominal aortic graft replacement was performed. The postoperative course was complicated by ureteral injury and graft infection, requiring redo graft replacement with omental coverage. The patient survived and remains alive at 57 years of age (Figure 1). During the clinical course, the patient experienced additional vascular complications, including a large right elbow hematoma caused by rupture of small distal arteries beyond the catheter access site and, on a separate occasion, a large femoral hematoma. Both events were treated conservatively.

**Figure 1:**
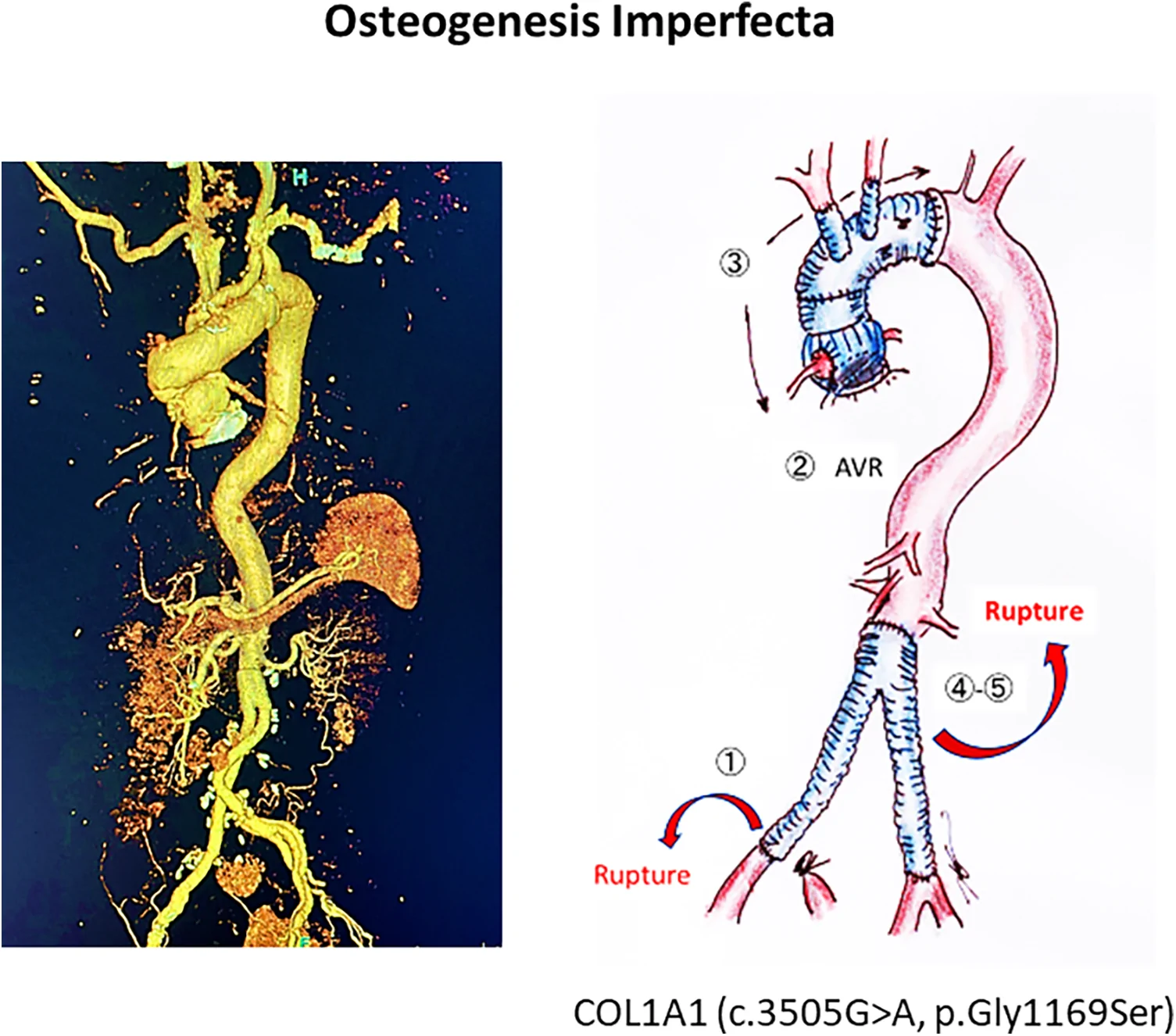
Imaging and schematic representation of vascular involvement in COL1A1-associated osteogenesis imperfecta. Three-dimensional CT angiography and corresponding schematic illustration demonstrate multilevel arterial involvement with dissection and rupture, including rupture at relatively small aortic diameters.

The patient had been treated with bisphosphonate (ibandronate). Bone metabolic markers demonstrated increased bone turnover (TRACP-5b 645 mU/dL, total PINP 210.2 ng/mL), with mildly reduced 25-hydroxyvitamin D (17.4 ng/mL) and a bone mineral density T-score of −2.4 SD.

Genetic testing identified a pathogenic COL1A1 variant (NM_000088.4: c.3505G > A, p. Gly1169Ser) affecting the triple-helical region of type I collagen.

### Case 2: FBN1-associated Marfan syndrome

A 60-year-old woman with clinically diagnosed Marfan syndrome, carrying an FBN1 splice-site variant (c.2677 + 5G > A), presented with a history of extensive aortic disease.

At 34 years of age, she developed Stanford type B aortic dissection during late pregnancy, requiring cesarean delivery followed by abdominal aortic graft replacement.

At 39 years of age, progressive enlargement of the thoracoabdominal aorta necessitated thoracoabdominal replacement with branch reconstruction.

At 42 years of age, she developed acute type A aortic dissection and underwent emergency total arch replacement with a frozen elephant trunk.

At 51 years of age, progressive dilation of the aortic root with mild to moderate aortic valve regurgitation required root replacement with a mechanical valved conduit.

At 52 years of age, coil embolization of a left internal thoracic artery aneurysm was performed (Supplementary Figure-A).

At 57 years of age, graft replacement was performed for dissected aneurysms involving the brachiocephalic artery, right common carotid artery, and right axillary artery (Supplementary Figure-B).

At 58 years of age, graft replacement was performed for dissected aneurysms involving the left subclavian, axillary, and brachial arteries (Figure 2).

**Figure 2:**
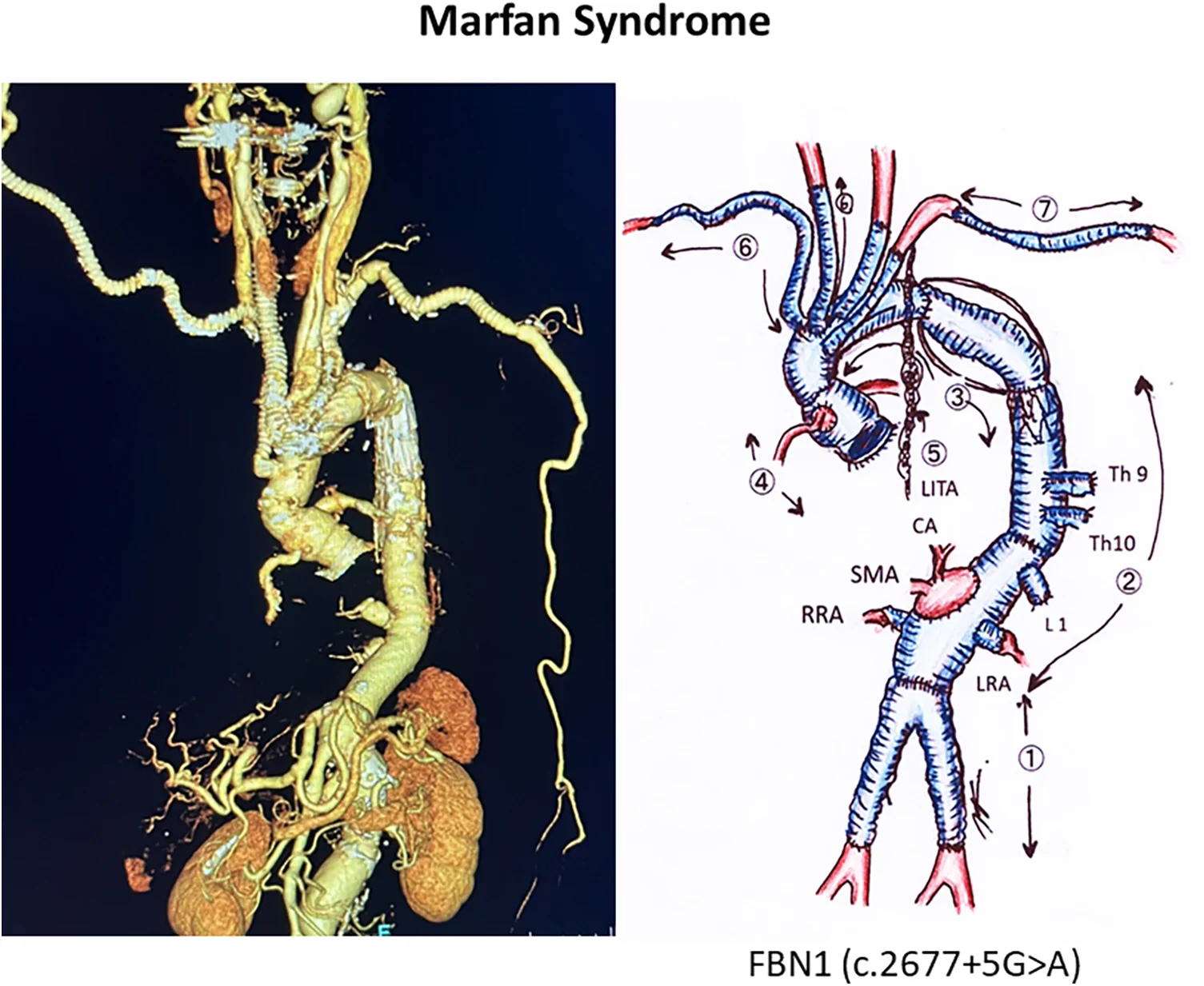
Imaging and schematic representation of vascular involvement in FBN1-associated Marfan syndrome. Three-dimensional CT angiography and corresponding schematic illustration demonstrate extensive arterial involvement characterized by dissection-driven progressive dilative remodeling affecting the aorta and branch vessels.

At present, residual dilatation of the bilateral extracranial internal carotid arteries measuring 10–15 mm and the vertebral arteries measuring approximately 10 mm is observed, and the patient is being followed up by the neurosurgery department (Supplementary Figure-C).

Genetic testing identified a splice-site variant in the FBN1 gene (NM_000138.5: c. 2677 + 5G > A) located in intron 21. No other pathogenic variants associated with arterial disease were detected.

## Histopathologic findings

Histopathologic examination of the aortic valve demonstrated comparable myxomatous degeneration in both cases, characterized by loosely arranged collagen fibers and reduced structural integrity (Figures 3A,B).

**Figure 3:**
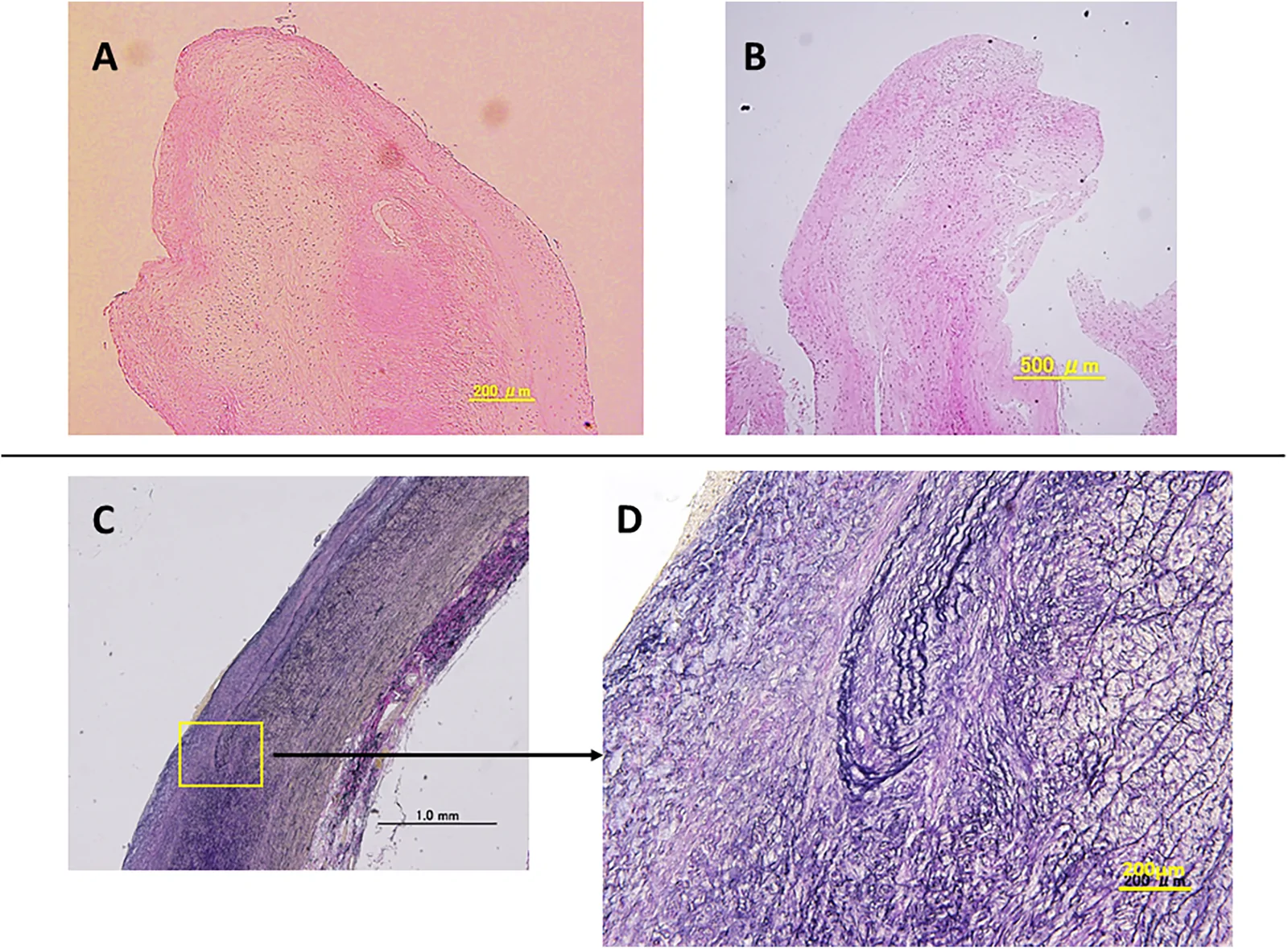
Histopathologic findings of the aortic valve in COL1A1- and FBN1-associated disease, and abdominal aorta in COL1A1-associated disease. **(A)** COL1A1-associated osteogenesis imperfecta showing marked myxomatous degeneration of the aortic valve with preservation of the overall layered architecture (Hematoxylin and eosin staining). **(B)** FBN1-associated Marfan syndrome showing similar myxomatous degeneration with preservation of the overall layered architecture (Hematoxylin and eosin staining). **(C)** Low-power view of a non-dissected abdominal aortic segment from the patient with COL1A1-associated osteogenesis imperfecta, demonstrating thinning of the aortic wall, with a minimum full-thickness measurement of approximately 1.25 mm (Elastica van Gieson staining). **(D)** Higher magnification demonstrates focal interruption of the internal elastic lamina together with fragmentation and attenuation of medial elastic fibers (Elastica van Gieson staining).

The abdominal aortic segment from the patient with COL1A1-associated OI demonstrated aortic wall thinning, with a minimum full-thickness measurement of approximately 1.25 mm, together with focal interruption of the internal elastic lamina and fragmentation of medial elastic fibers. Wall thickness was determined by concordant measurements obtained using both macroscopic ruler-based assessment of the gross specimen and calibrated microscopic scale analysis (Figures 3C,D).

Examination of the thoracic aortic wall revealed medial degeneration with fragmentation of elastic fibers in both patients (Figures 4A,B).

**Figure 4:**
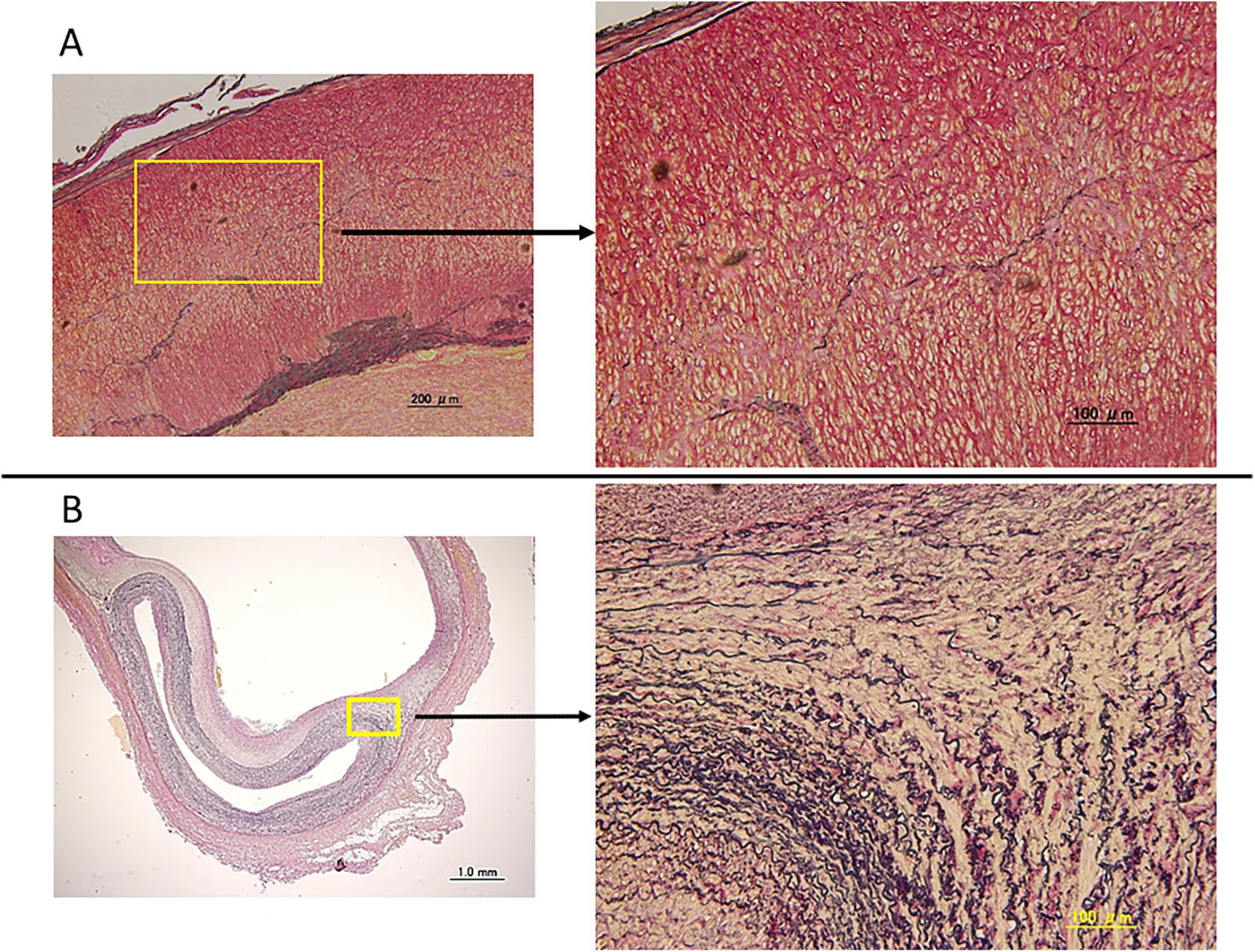
Histopathologic findings of the thoracic aortic wall in COL1A1-associated osteogenesis imperfecta and FBN1-associated Marfan syndrome. **(A)** COL1A1-associated osteogenesis imperfecta showing medial degeneration with fragmentation of elastic fibers and disruption of lamellar architecture (Elastica van Gieson staining). **(B)** FBN1-associated Marfan syndrome showing medial degeneration with fragmentation of elastic fibers (Elastica van Gieson staining).

## Discussion

The two patients presented here exhibited contrasting longitudinal vascular courses despite some overlapping histopathologic findings. Given that each genetic disorder is represented by a single patient, these observations should be interpreted as descriptive and hypothesis-generating rather than as evidence of disease- or genotype-specific patterns of vascular failure.

Balasubramanian et al. described rare but clinically significant cases of aortic aneurysm and dissection in OI, indicating that large-vessel involvement, while uncommon, can lead to severe complications (1).

In contrast, a nationwide cohort study reported that mortality due to cardiovascular diseases in patients with OI was not significantly increased compared to the general population, although overall survival was reduced in both males and females (2). In a subsequent nationwide register-based study by the same investigators, patients with OI demonstrated a significantly increased risk of valvular disease, including mitral (SHR 6.3) and aortic regurgitation (SHR 4.5), as well as heart failure and atrial fibrillation, whereas the risk of arterial aneurysms or dissections was not significantly increased (3). Consistent with these findings, a recent systematic review including 22 studies (12 case–control and 10 cohort/case series studies) demonstrated that cardiovascular abnormalities in OI are relatively common across all age groups and disease severities (4). Valvular abnormalities, predominantly mitral and aortic regurgitation, were reported in a wide range from 20.7% to 95% in adults and up to 37.7% in children, although many were mild or trivial. However, clinically significant disease requiring surgical intervention has also been described. Heart failure was more frequently observed, with earlier onset compared to controls, and echocardiographic studies suggested increased left ventricular dimensions and subtle reductions in systolic or diastolic function. In addition, increased aortic root dimensions were reported in several studies, with some patients exceeding a Z-score of >2, although the progression of these findings remains unclear (4).

Importantly, despite these widespread cardiovascular abnormalities, the prevalence of clinically overt arterial aneurysms or dissections was not significantly increased in population-based analyses, suggesting a discrepancy between structural vulnerability and clinically manifest large-vessel events.

The present COL1A1-associated patient experienced multiple arterial events, including rupture of the infrarenal abdominal aorta. Importantly, the rupture occurred in association with underlying aortic dissection rather than primary aneurysmal dilatation, and the aorta had measured only 30 mm in maximal diameter five months earlier. In the context of degenerative aneurysmal disease, rupture at such a diameter would be unusual. In this individual patient, therefore, vessel diameter alone did not reflect the subsequent occurrence of rupture.

Histopathologically, both cases exhibited medial degeneration with fragmentation of elastic fibers.

Medial degeneration and elastic fiber fragmentation are not specific to heritable connective tissue disorders and may occur in a variety of genetic and non-genetic aortic diseases. Notably, although established genetic aortopathies such as Marfan and Loeys–Dietz syndromes are included in the consensus classification of noninflammatory degenerative aortic disease, COL1A1-associated osteogenesis imperfecta is not specifically represented, and its aortic histopathologic features remain less well characterized (5).

Quantitative changes in the vascular wall may contribute to vascular fragility. Experimental studies provide additional insight into vascular fragility in COL1A1-related disease. In a murine model with deletion of the COL1A1 gene, decreased type I collagen content in the aortic wall was associated with fatal thoracic hemorrhage in more than half of the animals by 18 months of age (6) This finding suggests that vascular failure may occur as an acute event rather than a consequence of progressive enlargement, although the precise mechanism leading to rupture remains unclear. Further experimental work in mice using high-resolution magnetic resonance imaging demonstrated that no evidence of aneurysmal dilatation or prior dissection was observed before rupture, further supporting the concept that vascular failure may occur as an acute event rather than a consequence of progressive enlargement (7). Histologic analysis in the same study further revealed approximately 22% thinning of the cerebral arterial wall, supporting the concept that reduced wall thickness may contribute to decreased tensile strength and increased susceptibility to dissection and rupture (7). In the present case, the infrarenal abdominal aorta demonstrated a minimum full-thickness measurement of 1.25 mm, determined by concordant macroscopic and microscopic measurements, suggesting substantial structural attenuation of the vascular wall. The internal elastic lamina is composed primarily of elastin rather than type I collagen. In the present patient, focal interruption of the internal elastic lamina and disruption of medial elastic architecture were observed despite the primary genetic defect involving COL1A1. However, medial degeneration and elastic fiber abnormalities are not disease-specific, and the present histopathologic observations cannot establish whether or how the COL1A1 variant contributed to these changes. Similarly, the occurrence of multiple vascular events involving both large and small arteries is reported here as a feature of this individual clinical course and should not be interpreted as evidence of a generalized COL1A1-specific vascular phenotype.

In contrast, the FBN1-associated Marfan case, although lacking prominent physical features, demonstrated dissection-driven progressive dilative remodeling requiring repeated surgical interventions (8). Hung et al. identified 56 distinct FBN1 variants in 294 patients with Marfan syndrome. Among these, patients harboring the same variant as in the present case (c.2677 + 5G > A) exhibited similar clinical features, including a history of aortic disease and dural ectasia, with otherwise limited systemic manifestations (Supplementary Table) (9). According to the American College of Medical Genetics and Genomics (ACMG) criteria, this variant is currently classified as of uncertain significance; however, it may be associated with a relatively consistent phenotypic profile (10, 11).

Although genotype–phenotype correlations have been reported for FBN1 variants, substantial phenotypic heterogeneity remains, and no specific clinical manifestation can be reliably predicted from an individual variant alone. Therefore, the extensive vascular involvement observed in the present patient should be regarded as an individual clinical phenotype rather than a vascular pattern attributable specifically to the FBN1 variant (12). Given the progressive and extensive nature of aortic involvement in such cases, surgical management often requires repeated or extensive reconstruction. Estrera et al. reported outcomes of 39 patients who underwent complete aortic replacement among 3012 aortic surgeries performed between 1991 and 2013. Of these, 39% (14 of 39) had a history of connective tissue disorders, including Marfan syndrome in 9 patients, Loeys–Dietz syndrome in 1, and other disorders in 4. Among these 39 patients, 22 underwent aortic root replacement. The overall 20-year survival rate was 30% (13). In the present case, the patient remains alive and ambulatory 25 years after the initial surgery and continues regular outpatient follow-up. However, the timing of intervention for residual carotid artery lesions remains a clinical concern.

Although medial degeneration and elastic fiber fragmentation were observed in both patients, their longitudinal vascular courses differed. The FBN1-associated patient experienced repeated dissections followed by progressive vascular dilatation and extensive reconstruction, whereas the COL1A1-associated patient experienced rupture of a dissected infrarenal aortic segment that had remained relatively small in diameter. These observations illustrate the clinical heterogeneity encountered in these two patients but are insufficient to define disease- or genotype-specific patterns of vascular failure.

This report has several important limitations. It describes only two patients, one with each genetic disorder, and therefore cannot establish disease- or genotype-specific vascular patterns, incidence, or management thresholds. Vascular tissues were not systematically collected at each operation or from comparable vascular territories throughout the long clinical courses. Some relevant historical specimens are held at an outside institution, and the available archived specimens differ in age, preservation, sampling site, and amount of residual tissue, precluding reliable standardized longitudinal analysis. Age- and site-matched control tissues were not available. In addition, functional studies of the identified COL1A1 and FBN1 variants were not performed. Consequently, a causal relationship between genotype, histopathologic findings, and the observed vascular events cannot be established from these cases. Prospective tissue preservation, standardized longitudinal histopathologic assessment, appropriate controls, and functional analyses will be required to investigate these relationships in future patients.

## Conclusion

These two genetically defined patients exhibited contrasting longitudinal vascular courses. The COL1A1-associated OI patient experienced rupture of a dissected infrarenal aortic segment that had remained relatively small in diameter, whereas the FBN1-associated Marfan patient experienced repeated dissections followed by progressive vascular dilatation and extensive surgical reconstruction. Although no disease- or genotype-specific pattern or mechanism of vascular failure can be inferred from these two cases, their longitudinal clinical, imaging, and available histopathologic findings illustrate the heterogeneity of vascular manifestations in heritable connective tissue disorders. Further systematic investigation in additional patients will be required to determine whether these observations have broader genotype–phenotype significance.

## Data Availability

The clinical and genetic data supporting this study are derived from the patients' medical records maintained at Kyorin University Hospital. The relevant findings are presented in the article. The underlying patient-level data are not publicly available due to patient confidentiality and privacy restrictions.

## Supplementary material

Supplementary Figure Extensive peripheral arterial dilatation in the Marfan syndrome case. **(A)** Dilatation of the left internal thoracic artery before embolization. **(B)** Dilatation of the cervical branch vessels before graft replacement (posterior view). **(C)** Dilatation of the residual extracranial internal carotid and vertebral arteries. The internal carotid arteries are dilated to 10–15 mm in diameter, and the vertebral arteries are dilated approximately 10 mm.

Supplementary Table Clinical and Phenotypic Comparison Between COL1A1-Associated Osteogenesis Imperfecta and FBN1-Associated Marfan Aortopathy.
