# Supplementary material for: Distinct patterns of vascular failure in COL1A1-associated osteogenesis imperfecta and FBN1-associated Marfan syndrome
Source: Front Cardiovasc Med. 2026 Oct 27;13:1922704. doi: 10.3389/fcvm.2026.1922704 (PMC13748242; doi:10.3389/fcvm.2026.1922704)
Supplement: Supplementary file 1 [file Table1.docx]

Table

| Variable | COL1A1-Associated Osteogenesis Imperfecta | FBN1-Associated Marfan Syndrome |
| --- | --- | --- |
| Age at first aortic event | 36 years (aortic valve regurgetation and iliac artery rupture) | 34 years (Type B aortic dissection during pregnancy) |
| Age at latest follow-up | 57 years | 60 years |
| Gene | COL1A1 | FBN1 |
| Variant | c.3505G>A (p. Gly1169Ser) | c.2677+5G>A |
| Inheritance | Sporadic (no family history identified) | Family history suggestive of heritable aortic disease (no relatives with a formal diagnosis of Marfan syndrome) |
| Primary connective tissue defect | Type I collagen structural defect | Fibrillin-1 microfibril defect |
| Blue sclera, Genaralized joint laxity, Recurrent bone fractures, Dentinogenesis imperfecta, Hearing impairment | + | - |
| Skeletal system | Osteoporosis | No prominent skeletal abnormalities |
| Ocular system | Blue sclera | Normal |
| Dural ectasia | - | + |
| Cardiac manifestation | Severe aortic valve regurgitation without dilatation of the sinus of Valsalva | Mild to moderate aortic valve regurgitation with dilatation of the sinus of Valsalva |
| Valve tissue quality | Markedly fragile cusps at surgery | Generally preserved |
| Peripheral vascular involvement | Iliac artery dissection and rupture | Extensive dissection-related dilatation of branch vessels extending to the internal thoracic, subclavian, and cervical arteries, including the internal and external carotid arteries |
| Number of major vascular surgeries | Multiple staged procedures | Multiple staged procedures |
| Histopathology | Medial degeneration with fragmentation of elastic fibers Wall thinning and disruption of lamellar architecture of the abdominal aorta | Medial degeneration with fragmentation of elastic fibers |
| Structural failure pattern | Diameter-independent rupture / tissue fragility | Dissection with subsequent dilative remodeling |
